# Generation of Antibodies Selectively Recognizing Epitopes in a Formaldehyde-Fixed Cell-Surface Antigen Using Virus-like Particle Display and Hybridoma Technology

**DOI:** 10.3390/antib12030057

**Published:** 2023-09-05

**Authors:** Stefanie Schatz, Lena Willnow, Monika Winkels, Jamila Franca Rosengarten, Benjamin Theek, Ian C. D. Johnston, Jörn Stitz

**Affiliations:** 1 Research Group Medical Biotechnology and Bioengineering, Faculty of Applied Natural Sciences, University of Applied Sciences Cologne, Campusplatz 1, 51379 Leverkusen, Germany; 2 Institute of Technical Chemistry, Gottfried Wilhelm Leibniz University Hannover, Calinstrasse 3-9, 30167 Hannover, Germany; 3 Miltenyi Biotec B.V. & Co. KG, Friedrich-Ebert-Strasse 68, 51429 Bergisch Gladbach, Germany

**Keywords:** antibody discovery, virus-like particles, cell-surface antigen, antigen display, hybridoma technology, nerve growth factor receptor, formaldehyde-fixed paraffin-embedded (FFPE) tissue samples

## Abstract

Efficient induction of target-specific antibodies can be elicited upon immunization with highly immunogenic virus-like particles (VLPs) decorated with desired membrane-anchored target antigens (Ags). However, for example, for diagnostic purposes, monoclonal antibodies (mAbs) are required to enable the histological examination of formaldehyde-fixed paraffin-embedded (FFPE) biopsy tissue samples. Aiming at the generation of FFPE-antigen-specific mAbs and as a proof of concept (POC), we first established a simplified protocol using only formaldehyde and 90 °C heat fixation (FF90) of cells expressing the target Ag nerve growth factor receptor (NGFR). The FF90 procedure was validated using flow cytometric analysis and two mAbs recognizing either the native and FFPE-Ag or exclusively the native Ag. C-terminally truncated NGFR (trNGFR)-displaying native and FF90-treated VLPs derived from HIV-1 did not reveal distinctive changes in particle morphology using transmission electron microscopy (TEM) and dynamic light scattering (DLS) analysis. Mice were subsequently repetitively immunized with trNGFR-decorated FF90-VLPs and hybridoma technology was used to establish mAb-producing cell clones. In multiple screening rounds, nine cell clones were identified producing mAbs distinctively recognizing epitopes in FF90- and FFPE-NGFR. This POC of a new methodology should foster the future generation of mAbs selectively targeting FFPE-fixed cell-surface Ags.

## 1. Introduction

In 1975, Köhler and Milstein developed a new method involving the fusion of antibody-producing primary cells and immortalized cells (hybridoma technology) to generate monoclonal antibodies (mAbs) [1]. Typically, mice are repeatedly immunized with the target antigen (Ag) prior to sacrifice and the isolation of B cells from the spleen. To render these primary cells accessible to long-term culture in vitro, they are subsequently fused with transformed myeloma cells to form hybridoma cells. Although cell fusion occurs only at low efficiencies, sufficient numbers of cell clones are generated to identify a range of Ag-reactive hybridomas resulting from the overrepresentation of Ag-specific B cells in the donor’s blood upon immunization. Hits, i.e., clones producing Ag-specific mAbs, are identified in one or multiple screening rounds for binding against target and non-target Ags employing enzyme-linked immunosorbent assay (ELISA) or flow cytometry. Isolated hybridoma cell clones can subsequently also be employed for the production of the cognate mAbs on a larger scale [2].

While the utilization of selected murine mAbs as therapeutic active substances is often limited due to their immunogenicity in human recipients, they can be applied to the specific detection and quantitation of the target Ags enabling the development of, e.g., ELISA, flow cytometric and Western blot analysis protocols instrumental for research and diagnostic purposes. In addition to these applications, mAbs coupled to fluorophores or haptens are valuable tools in the detection of antigens in histological preparations. Frequently and to facilitate long-term preservation, such tissue samples are formaldehyde-fixed and paraffin-embedded (FFPE) [3]. Unfortunately, most mAbs developed against native Ags fail to recognize and bind the FFPE-treated molecules owing to the altered structure of the target epitopes upon fixation [4,5]. During the FFPE procedure proteins are subjected to formaldehyde fixation, dehydration in alcohol solutions, the solvent xylene and temperatures between 50 °C and 70 °C during paraffin embedding. As a consequence, the FFPE treatment leads to chemical modifications of Ags and cross-linking of proteins induced by formaldehyde as well as protein aggregation and denaturation [6,7]. Consequently, new epitopes are generated and epitopes that were accessible in the native Ag are masked reducing or blocking the immunoreactivity of mAbs designed for native Ags [4]. Thus, methodologies aiming at the specific generation of FFPE-Ag-specific mAbs are required.

The utilization of soluble Ags such as secreted or cytoplasmic proteins for the immunization of mice to initiate hybridoma-based antibody induction and selection can be conducted in a straightforward manner. However, when membrane-anchored surface-Ags are intended to be used, hydrophobic transmembrane regions (TMRs) mount major challenges. The simple truncation of a single membrane passaging protein deleting the TMR and intracellular part leaving only the yet soluble ectodomain may also alter its three-dimensional structure, hampering the elicitation of mAbs recognizing the full-length target protein. Obviously, this issue increases with the number of TMRs. One way to circumvent this is the display of cell-surface proteins on membrane-enveloped virus-like particles (VLPs) [8,9]. VLPs can be decorated with heterologous viral and non-viral proteins. VLPs are highly immunogenic eliciting, amongst others, a strong B cell response [10,11].

We hypothesized that VLPs displaying heterologous cell-surface Ags at considerable densities and upon FFPE-like fixation, could be instrumental in hybridoma technology to generate FFPE-Ag-specific mAbs. A C-terminally truncated variant of the human low-affinity nerve growth factor receptor (trNGFR) was chosen as a model Ag in this proof of concept (POC) study [12,13] and VLP-producing human suspension cell pools were established expressing HIV-1 Gag [14] and trNGFR [15,16]. As the preparation of FFPE-fixed VLPs is very challenging and paraffin embedding of VLPs is not possible, we developed a simplified fixation procedure. To establish the protocol mimicking FFPE fixation, we tested a range of conditions using 293-F cells expressing trNGFR and two mAbs recognizing either the native target antigen or both, the native and FFPE-target Ag. The identified novel protocol was employed to fix trNGFR-VLPs and subsequently used to immunize mice. Splenocytes were isolated and fused to form hybridoma cells. Secreted mAbs were screened for reactivity against native and fixed target cells. Using the novel simplified fixation protocol for the preparation of target Ag-displaying VLPs, nine clones were identified producing mAbs selectively binding FFPE-NGFR target Ag.

## 2. Materials and Methods

### 2.1. Plasmids

An overview of the design of the expression constructs used to generate the recombinant 293-F VLP and trNGFR-VLP producer cells as well as the trNGFR-expressing murine screening cell line can be found in Figure S1. The transposase expression vectors pCMV-SB100x and pCMV-hyPBase mediate the expression of the hyperactive *Sleeping Beauty* variant SB100x [12] and the hyperactive piggyBac (hyPBase) transposase (GenBank Accession No. OL519599.1, [13,14]), respectively. Expression in both transposon vector constructs is driven by the cytomegalovirus promoter/enhancer (P_CMV_). The expression of the transposases and the two reporter genes truncated nerve growth factor receptor (*trngfr*) [15,16] and secreted alkaline phosphatase (*seap*; GenBank Accession No. U89938, Clontech Laboratories, Mountain View, CA, USA), are coupled to each other by separating synthetic introns (IVS) and internal ribosomal entry sites (IRES).

The HIV-1 Gag expression cassette in PB transposon donor vector pPB-mos1.gag-IpW is flanked by PB-derived inverted terminal repeats (ITRs) and the expression of the HIV-1 mosaic 1 Gag proteins (Mos1.Gag) [17] is coupled by an IVS and an IRES to a puromycin resistance gene (*puro^R^*) serving as a selectable marker [18]. The woodchuck hepatitis virus posttranscriptional element (WPRE) enhances the half-life of transcripts. The downstream located bovine growth hormone polyadenylation signal (BGH p(A)) terminates the expression cassette. The details of the cloning strategy of the transposase expression vectors pCMV-SB100x and pCMV-hyPBase and the PB transposon donor vector pPB-mos1.gag-IpW have been described previously [18,19,20].

The SB transposon donor vector pSB-cHS4-trNGFR-IhW vector encodes for the C-terminally truncated NGFR variant (trNGFR) encompassing a cytoplasmic domain of only 8 amino acids [15,16], while full-length NGFR entails 155 residues in this region. The pSB-cHS4-trNGFR-IhW was constructed by amplification of *trngfr* PCR fragments from pCMV-SB100x using the primer pair NheI-trNGFR forward (5′-ATA TGC TAG CAC CAT GGG GGC AGG TGC CAC-3′, Eurofins Genomics, Ebersberg, Germany) and SalI-trNGFR reverse (5′-ATA TGT CGA CCT AGA GGA TCC CCC TGT TCC AC-3′, Eurofins Genomics, Ebersberg, Germany). The ampflified *trngfr* DNA fragments and the recipient vector were digested with the enzymes indicated in the primer names and the insert ligated into the opened pSB-cHS4-IhW recipient vector. Restriction enzyme analysis and Sanger sequencing (Microsynth Seqlab, Göttingen, Germany) of the *trngfr* coding sequence confirmed the successful insertion of *trngfr* into the recipient vector. In the recipient vector, the SB-derived ITRs flank two core chicken beta-globin insulator sequences at the 5′- and 3′-end of the cassette (cHS4) [21] shielding the P_CMV_ from promoter silencing. The DNA sequences of cHS4 (GenBank Accession No. U78775), the SB-ITRs originating from the Tc-like element (GenBank Accession No. L48685; [20,22]) and the P_CMV_ were synthesized and inserted into a pUC57 plasmid (GenScript, Piscataway, NJ, USA) to generate pSB-cHS4. The IVS-IRES-hygro^R^ fragment was derived from the pIREShyg3 (Clontech Laboratories, Mountain View, CA, USA) and inserted into the pSB-cHS4 plasmid to generate the pSB-cHS4-IhW recipient vector. Cloning strategy details are available upon reasonable request.

### 2.2. Cells

Murine NIH/3T3 fibroblast cells (ATCC Cat. No. CRL-1658, RRID: CVCL_0594) were cultivated in Dulbecco’s modified Eagle pyruvate medium high glucose (DMEM; Gibco/Thermo Fisher Scientific, Waltham, MA, USA) supplemented with 4 mM L-glutamine and 10% fetal bovine serum (FBS; Gibco/Thermo Fisher Scientific, USA). Cell cultures were expanded at 37 °C in a humidified atmosphere at 5% CO_2_. Adherent cells were detached using 1 mM ethylenediaminetetraacetic acid (EDTA; VWR International, Radnor, PA, USA) in phosphate-buffered saline (PBS; Gibco/Thermo Fisher Scientific, Waltham, MA, USA) for passaging.

Sp2/0-Ag14 mouse myeloma cells (ATCC Cat. No. CRL-8287, RRID: CVCL_2199) were cultivated in DMEM (BioWest, Nuaillé, France) supplemented with 10% HyClone FetalClone I FBS (Cytiva, Marlborough, MA, USA), 2 mM L-glutamine (Gibco/Thermo Fisher Scientific, Waltham, MA, USA) and 20 mM HEPES (Lonza, Basel, Switzerland) at 37 °C in a humidified atmosphere at 9% CO_2_.

Human Freestyle 293-F suspension cells (RRID: CVCL_D603; Thermo Fisher Scientific, USA) were cultured in serum-free Freestyle Expression medium (Thermo Fisher Scientific, USA) at 37 °C, 8% CO_2_ and 135 rpm in a shaker incubator (Minitron, Infors HT, Switzerland). Cells were passaged at viable cell densities (VCDs) between 0.3 × 10^6^ and 2.0 × 10^6^ viable cells/mL.

### 2.3. Establishment of VLP Producer Cells and Murine Cells Expressing Human trNGFR

The stable 293-F VLP producer cell pool 293-F/Mos1.Gag was generated using the PB transposon vectors pCMV-hyPBase and pPB-mos1.gag-IpW as previously described [18]. The stable 293-F trNGFR-VLP producer cell pool 293-F/Mos1.Gag/trNGFR was subsequently established utilizing SB-mediated transposon gene transfer [19]. Then, 16.0 μg of pSB-trNGFR-IhW and 2.5 μg of pCMV-SB100x were incubated with 110 μg PEI transfection reagent (Polysciences Inc, Warrington, PA USA) and co-transfected into 30 × 10^6^ viable 293-F/Mos1.Gag cells/mL. Three days post-transfection, stable cells were initially selected using 50 μg/mL hygromycin (InvivoGen, Toulouse, France). Hygromycin concentrations were constantly elevated for three weeks to a final concentration of 200 μg/mL. For further expansion, 10 μg/mL puromycin (InvivoGen, France) and 200 µg/mL hygromycin were constantly applied. 

3T3/Gag/trNGFR cells were used in screening for reactive hybridoma clones. Briefly, 10^5^ NIH/3T3 cells stably expressing wild-type HIV-1 Gag of molecular clone NY5 (Uniprot Accession No. P12493) were seeded in a 6-well plate (Greiner Bio-One, Kremsmünster, Austria). On the following day, cells were co-transfected using 0.25 µg of pCMV-SB100x and 2.25 µg of pSB-chS4-trNGFR-IhW vectors diluted in OptiMEM I medium (Thermo Fisher Scientific, USA) and the TransIT^®^ LT-1 transfection reagent (Mirus Bio, Madison, WI, USA). Stably transfected cells were selected using increasing concentrations of up to 400 µg/mL hygromycin. The screening cell line was generated from the pre-selected 3T3/Gag/trNGFR cell pool by sorting and collecting the top 15% of trNGFR-expressing cells using flow cytometry (see Section 2.6) and subsequent limiting dilution to generate single-cell clones. After re-assessing the trNGFR expression using flow cytometry measurement, a single-cell clone with high trNGFR expression was chosen.

### 2.4. VLP Preparation

293-F VLP and trNGFR-VLP producer cells were seeded at 0.5 × 10^6^ viable cells/mL in 20 mL serum-free medium and expanded for three days. The viabilities of the cell cultures were determined three days after inoculation using a cell counter (anvajo GmbH, Dresden, Germany). VLPs were only harvested from cultures revealing a viability above 90%. To harvest VLPs, supernatants were separated from the cells using low-speed centrifugation at 300 rcf for 5 min. The clarified supernatants were passaged through 0.45 μm polyvinylidene fluoride (PVDF) membrane syringe filters (Carl Roth, Karlsruhe, Germany) to remove contaminating cells and cell debris. Cell-free supernatants (CFSNs) were either stored at −20 °C or subjected to a concentration of VLPs employing ultracentrifugation at 112,700 rcf and 4 °C for 1.5 h using an Optima XE-90 ultracentrifuge (Beckman Coulter, USA), SW28 swing-out rotor (Beckman Coulter, Brea, CA, USA) and Ultra Clear centrifuge tubes (Beckman Coulter, Brea, CA USA). The supernatants were discarded and air-dried VLP pellets were resuspended in 60 μL storage solution (10% (*w*/*v*) trehalose dissolved in PBS [23]) per 10 mL CFSN and stored at −80 °C.

### 2.5. Fixation of Cells and VLPs

Cells were washed with PBS and fixed using a 4% formaldehyde solution (Carl Roth, Karlsruhe, Germany) for 1 h. Formaldehyde-fixed cells were pelleted by centrifugation at 300 rcf for 5 min and washed three times in PBS. Formaldehyde-fixed cells were suspended in PBS and subjected to heat fixation at 70 °C, 80 °C, 90 °C and 100 °C for 1 h, respectively. After cooldown, cells were stored in PBS at 4 °C.

Formaldehyde-fixed cells were incubated in increasing concentrations of ethanol without exchange of the ethanol solution between each step. 1 × 10^6^ cells were initially incubated in 2 mL 48% ethanol for 20 min. An additional 2 mL of 99.8% ethanol (Carl Roth, Karlsruhe, Germany) was added to a final ethanol concentration of approximately 70%. Cells were incubated for another 20 min. Finally, an additional 15 mL of 99.8% ethanol was added and cells were incubated for 20 min. The resulting formaldehyde- and ethanol-fixed cells were pelleted by centrifugation at 600 rcf for 5 min, washed and stored in PBS.

Formaldehyde-fixed paraffin-embedded (FFPE) cells were prepared according to an internal, confidential protocol of Miltenyi Biotec, Bergisch Gladbach, Germany. The procedure mimics the steps performed during FFPE tissue embedding. Cells were first fixed using a 4% paraformaldehyde solution. Dehydration was performed using ascending ethanol series, clearing in xylene substitute (ROTI^®^Histol; Carl Roth, Karlsruhe, Germany) and embedding in paraffin at 60 °C. Cells were deparaffinized and rehydrated utilizing a descending ethanol series. Heat-induced antigen retrieval was conducted using a Tris-EDTA-citrate buffer (Miltenyi Biotec, Bergisch Gladbach, Germany). Fixed cells were stored at 4 °C in AutoMACS Running Buffer (Miltenyi Biotec, Bergisch Gladbach, Germany).

To fix VLPs using formaldehyde and 90 °C-treatment (FF90), VLPs pooled from multiple cultivations and containing up to 2 µg Gag proteins in total were fixed in 2 mL 4% formaldehyde solution for 1 h. The reaction was terminated by adding 150 mM glycine. VLP samples were diluted in 35 mL PBS and pelleted by ultracentrifugation as described above. The pellets were resuspended in up to 2 mL PBS, heated to 90 °C for 1 h, sterile-filtered using 0.45 μm PVDF syringe filters (Carl Roth, Germany) and stored at −80 °C.

### 2.6. Flow Cytometric Analysis during Fixation Protocol and Screening Cell Line Development

Up to 1 × 10^6^ 293-F/Mos1.Gag/trNGFR or 3T3/Gag/trNGFR cells were washed in PBS and resuspended in 100 µL flow cytometry (FC) buffer (PBS supplemented with 2 mM EDTA and 0.5% (*w*/*v*) BSA) with dilutions of the phycoerythrin (PE)--conjugated anti-NGFR mAbs REA844 (RRID: AB_2725864; Miltenyi Biotec, Bergisch Gladbach, Germany) and C40-1457 (RRID: AB_396599; BD Biosciences, Franklin Lakes, NJ, USA), respectively. For antibody binding, cells were incubated in the dark at 4 °C for 10 min. Cells immunolabelled with matching isotype control antibodies served as negative controls (human isotype control mAb REA293, RRID: AB_2733893; Miltenyi Biotec, Germany; murine isotype control mAb MOPC-21, RRID: AB_396514; BD Biosciences, USA). Subsequently, cells were washed in 1 mL PBS, pelleted at 300 rcf for 5 min and resuspended in 1 mL FC buffer. Flow cytometric analysis was performed using a S3e Cell Sorter (Bio-Rad, USA). Data analysis was performed using the software FlowJo v10 (BD Biosciences, Franklin Lakes, NJ, USA) or MACSQuantify (Miltenyi Biotec, Bergisch Gladbach, Germany). The mean fluorescent intensity (MFI) and the standard deviation (SD) were calculated using the software FlowJo (BD Biosciences, USA). The stain index (SI) was calculated as follows: MFI_iso_ of the negative population stained with isotype control antibodies was subtracted from the MFI_sample_ of the cell population stained with the antigen-specific antibodies divided by two times the SD_iso_ of the isotype control cells [24].

SI = (MFI_sample_ − MFI_iso_)/(2 × SD_iso_) (1)

### 2.7. Protein Quantitation

To quantify the total protein amount in FF90-trNGFR-VLP preparations for injection, the ROTI^®^Nanoquant reagent (Carl Roth, Karlsruhe, Germany) was used according to the manufacturer’s instructions. Dilutions of bovine serum albumin (BSA; Carl Roth, Germany) in H_2_O were employed for calibration according to the instructions for the ROTI^®^Nanoquant assay. The optical densities of the standard dilutions and samples in 96-well plates (Brand, Wertheim, Germany) were examined at 590 nm and 450 nm using the Inifinite M1000Pro microplate reader (Tecan, Männedorf, Switzerland). 

Analytical duplicates of CFSN and VLP pellets were analyzed for Gag and NGFR concentrations using commercially available ELISAs and the microplate reader Multiskan FC (Thermo Fisher Scientific, USA). Precursor p55-Gag proteins were detected utilizing the QuickTiter™ HIV Lentivirus Quantitation Kit (HIV p24 ELISA; Cell Biolabs, San Diego, CA, USA). The p24-Gag standard dilution series was changed to cover a concentration range of 0.39 ng/mL to 25 ng/mL. Apart from this, the p24 ELISA was performed according to the manufacturer’s instructions. VLP particle numbers were calculated under the assumption that one VLP consists of an average of 3500 p24-Gag molecules as a subunit in the p55-Gag precursor [25] and a molecular weight of p24-Gag of 24,000 Da (used for calibration). This equates to 7.17 × 10^9^ VLPs per 1 μg p24-Gag.

NGFR concentrations were assessed employing the RayBio^®^ Human NGF R ELISA Kit following the manufacturer’s instructions (RayBiotech, Peachtree Corners, GA, USA). The molecular weight of trNGFR was approximated to be 29,500 Da using the ProtParam Tool (RRID: SCR_018087) as based on the amino acid sequence. The number of trNGFR molecules in one VLP was calculated using Equation (2):

n_trNGFR_ = (N_A_ × m_trNGFR_ × n_VLP_)/(M ×F), (2)

with n_trNGFR_ as the number of trNGFR molecules per VLP; m_trNGFR_ as the total amount of trNGFR per 1 µg p24-Gag; N_A_ as the Avogadro constant; n_VLP_ as the number of VLPs per 1 µg p24-Gag; M as the molecular weight of trNGFR and F as the conversion factor of 10^9^ ng/g.

### 2.8. VLP Capture Assay

To assess the incorporation of NGFR variants into HIV-derived particles, the VLP capture assay was conducted as previously reported [26]. In brief, protein A-conjugated magnetic beads (Dynabeads Protein A immunoprecipitation kit; Thermo Fisher Scientific, Waltham, MA, USA) were incubated with 10 μg anti-NGFR mAb REA844 (Miltenyi Biotec, Bergisch Gladbach, Germany) and human IgG isotype control antibodies (RRID: AB_2532958; Thermo Fisher Scientific, Waltham, MA, USA), respectively, under constant rotation for 1 h at room temperature (RT). Afterwards, the antibody-coated beads were washed extensively to remove unbound antibodies. Antibody-coated beads were subsequently incubated with 25 ng of VLPs for 3 h under rotation. Again, the beads were washed thoroughly to remove unbound VLPs. To dissociate captured VLPs and antibodies from the beads and to prepare denatured protein samples, beads were incubated with 22.5 μL elution buffer (Thermo Fisher Scientific, Waltham, MA, USA) mixed with 7.5 μL 4 × ROTI Load Laemmli buffer (Carl Roth, Karlsruhe, Germany) for 5 min at 95 °C. Using magnets, beads were separated from denatured proteins. 15 μL of each denatured protein sample were subjected to sodium dodecyl sulfate-polyacrylamide gel electrophoresis (SDS-PAGE) and subsequent Western blot analysis using anti-HIV-Gag p17 p24 p55 polyclonal rabbit antibodies (RRID: AB_1139524; Abcam, Cambridge, UK) diluted 1:2000 in TBS-T (2 mM Tris-HCl (Carl Roth, Karlsruhe, Germany), 15 mM NaCl (Carl Roth, Karlsruhe, Germany), 0.05% (*v*/*v*) Tween-20 (Carl Roth, Karlsruhe, Germany); pH 7.4). Polyclonal chicken anti-rabbit IgG-horseradish peroxidase (HRP) conjugates (RRID: AB_2534661; Thermo Fisher Scientific, Waltham, MA, USA) diluted 1:5000 in TBS-T served as secondary antibodies. Chemiluminescence detection (SuperSignal™ West Pico PLUS chemiluminescent substrate, Thermo Fisher Scientific, Waltham, MA, USA; ChemiDoc imaging system, Bio-Rad, Hercules, CA, USA) was employed to visualize labeled p55-Gag proteins.

### 2.9. Dynamic Light Scattering

Particle size analysis was performed using a Zetasizer nano ZS (Malvern Panalytical, Malvern, UK) and quartz glass cuvettes with an optical path length of 10 mm and 4 mm width, respectively. A volume of 100 μL of concentrated native or fixed VLP samples were used and backscatter was detected at 173°. For each sample, 15 runs were conducted for a duration of 10 s using the model “protein analysis“ (solvent: water, refractive index of 1.330, viscosity of 0.8872 mPa s; material: protein, refractive index of 1.450).

### 2.10. Transmission Electron Microscopic Analysis

VLPs were visualized using uranyl acetate negative staining method and subsequent transmission electron microscopy (TEM). VLP preparation and TEM imaging were performed by the Imaging Facility Services at CECAD, Cologne, Germany. Briefly, VLPs resuspended in PBS or storage solution were mixed with paraformaldehyde in PBS at a final concentration of 1%. Volumes of 5 μL were transferred to copper grids. Upon 20 min incubation at RT, grids were repeatedly washed with PBS and incubated with 1% (*v*/*v*) glutaraldehyde in PBS. Samples were again washed extensively with H_2_O and stained with 1% (*w*/*v*) uranyl acetate solution for 4 min at RT in the dark. Air-dried samples were visualized utilizing a 200 kV JEM-2100Plus Electron Microscope (JEOL, Freising, Germany).

### 2.11. Statistical Analysis

The OriginPro software version 2022b (OriginLab Corporation, Northampton, MA, USA) was employed for statistical analysis. To determine *p*-values and to assess the mean difference between the two samples, an independent two-sample t-test was applied. *p*-values of <0.05 were considered as statistically significant. In addition, to determine whether two samples revealed equal variance, a two-sample test for variance was used. The Welch’s correction for *t*-tests was used for two samples differing in variance at the 0.05 level. 

### 2.12. Immunization of Mice

**Figure 1:**
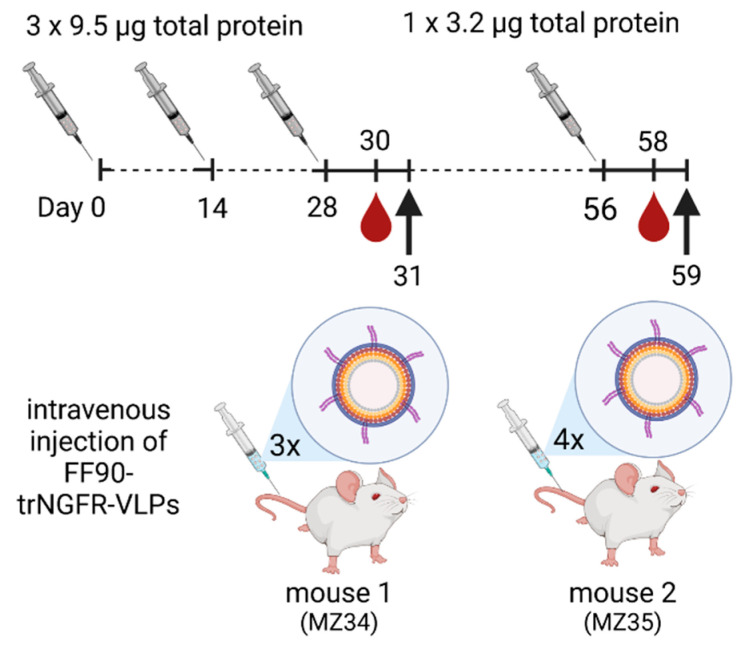
Schematic illustration of the immunization schedule. Mice were immunized on days 0, 14 and 28 with doses of 9.5 µg total protein of FF90- trNGFR-VLPs. On day 56, mouse 2 (MZ35) received a fourth immunization with 3.2 µg total protein of FF90-trNGFR-VLPs. Mice were bled two days after the last dose, hybridoma cell fusion was performed on the following day (indicated by the arrow) and blood samples were analyzed for the presence of NGFR-specific antibodies. Created with BioRender.com (accessed on 20 July 2023).

Two female 8-week-old BALB/cAnNCrl mice (RRID: IMSR_CRL:547; Charles River, Germany) were immunized intravenously with sterile-filtered 9.5 μg total protein of FF90-trNGFR-VLPs in PBS on days 0, 14 and 28. Two days after the last injection, blood samples were taken and analyzed for reactive antibodies (see Section 2.14). One mouse was sacrificed and splenocytes were subjected to hybridoma cell generation. On day 56, the second mouse received a fourth immunization with 3.2 μg total protein of FF90-trNGFR-VLPs. Two days after the last injection, blood samples were taken and analyzed for reactive antibodies. Mouse 2 was sacrificed and splenocytes were subjected to hybridoma cell generation. An overview of the immunization schedule is shown in Figure 1.

### 2.13. Generation of Hybridoma Cells

Murine Sp2/0-Ag14 myeloma cells were fused with isolated splenocytes using polyethylene glycol. Generated hybridoma cells were seeded into flat bottom 96-well cell culture plates (Greiner Bio-One, Kremsmünster, Austria) and expanded in hypoxanthine- and azaserine-containing HAT selection medium based on DMEM supplemented with 2 mM L-glutamine, 20 mM HEPES and 20% FBS. Five days after fusion, cells were fed with growth medium (DMEM, 2 mM L-glutamine, 20 mM HEPES, 20% FBS and 0.1 mM hypoxanthine). Wells revealing one or more cell clusters were analyzed for antigen-specific antibody expression. While positive single-cell clusters were directly expanded, reactive cultures showing multiple cell clusters were subjected to biological cloning to establish monoclonal cell cultures.

### 2.14. Screening for Antibodies

Flow cytometric analysis using native, FF90- and FFPE-fixed 3T3/Gag/trNGFR cells and human peripheral blood mononuclear cells (PBMCs) was conducted to identify NGFR-specific mAbs. PBMCs were isolated from buffy coats of healthy anonymous donors purchased from the German blood donation center (Deutsches Rotes Kreuz, Hagen, Germany). Prior to the exposure to mAbs, PBMCs were first incubated with a human Fc receptor (FcR) blocking reagent for 10 min at RT (Miltenyi Biotec, Bergisch Gladbach, Germany). 3T3/Gag/trNGFR cells were blocked using murine Fc receptor (FcR) blocking reagent for 10 min at RT (Miltenyi Biotec, Bergisch Gladbach, Germany). 

To detect NGFR-specific mAbs potentially elicited in immunized mice, blood samples were centrifuged at 1000 rcf for 10 min and 25 μL to 35 μL of serum were incubated with 2 × 10^5^ native cells and 3 × 10^5^ FF90-3T3/Gag/trNGFR cells, respectively, for 20 min at RT. The serum was examined for murine IgG antibodies binding to 3T3/Gag/trNGFR cells employing the PE-conjugated anti-mouse IgG1 and IgG2ab secondary mAbs X-56 and X-57 (RRID: AB_2751742 and AB_2733867; Miltenyi Biotec, Bergisch Gladbach, Germany). The secondary antibodies and the 3T3/Gag/trNGFR cells were incubated for 15 min at RT. 

To identify NGFR-reactive hybridoma mAbs, 85 μL supernatants harvested from cell clones were incubated with 2 × 10^4^ native and fixed cells, respectively, for 20 min at RT. Cells were washed twice with FC buffer. Subsequently, cells were incubated with PE-conjugated anti-mouse IgG1 mAb X-56 (RRID: AB_2751742; Miltenyi Biotec, Bergisch Gladbach, Germany) and APC-conjugated anti-mouse IgG2ab mAb X-57 (RRID: AB_2733867; Miltenyi Biotec, Bergisch Gladbach, Germany) for 15 min at RT. Cells were washed again, resuspended in 50 μL FC buffer and examined using a MACSQuant Analyzer 10 flow cytometer (Miltenyi Biotec, Bergisch Gladbach, Germany). Cells incubated with either DMEM or 15 ng of anti-NGFR antibody C40-1457 (RRID: AB_2152663; BD Biosciences, Franklin Lakes, NJ, USA) and secondary antibody conjugates served as negative and positive controls, respectively. Native and fixed cells were discriminated in a VioBlue versus FSC-A plot. Fixed cells showed autofluorescence at 450/50 nm (VioBlue channel), and thus could be distinguished from native cells. 

## 3. Results and Discussion

### 3.1. Characterization of VLP Producer Cell Pools

Stable human 293-F/Mos1.Gag VLP producer cells were established as previously described mediating the formation of bald VLPs [18]. These cells were co-transfected with the transposase expression construct pCMV-SB100x and the transposon donor vector pSB-trNGFR-IhW encoding for a truncated human nerve growth factor receptor (trNGFR) variant to establish the stable 293-F/Mos1.Gag/trNGFR cell pool. 

The formation of VLPs and the incorporation of the trNGFR into VLPs were first assessed using Gag- and NGFR-specific ELISAs. Cell-free supernatants (CFSNs) were harvested from cell cultures and investigated for their p24-Gag content. CFSNs of 293-F/Mos1.Gag cells contained on average 180 ± 59 ng/mL p24-Gag. In VLP-containing CFSNs of 293-F/Mos1.Gag/trNGFR cells, 53 ± 7 ng/mL p24-Gag were detected. Gag protein was not detectable in CFSNs of 293-F cells. Subsequently, CFSNs of VLP producer cell pools were normalized to a concentration of 45 ng/mL p24-Gag and NGFR concentrations were investigated by conducting an NGFR-specific ELISA. Although parental 293-F cells expressed wild-type NGFR [27], no NGFR was detectable in the CFSN of 293-F cells (Table 1). Thus, wild-type full-length NGFR or its degradation products were not secreted in notable amounts (Table 1). NGFR was also not observed in CFSNs of the VLP producer cell pool 293-F/Mos1.Gag strongly indicating that ectopically expressed wild-type NGFR is not incorporated into HIV-derived VLPs (Table 1). As wild-type NGFR is not excluded from lipid rafts [28], the sites of HIV particle budding, it is likely to assume that the failure to incorporate wild-type NGFR in VLPs is due to steric hindrance resulting from the long cytoplasmic domain of 155 amino acids. 

In contrast, trNGFR, with a truncated cytoplasmic tail of only 8 amino acids, was readily detected in CFSNs harvested from 293-F/Mos1.Gag/trNGFR cells. This gave a first indication that the detected antigen was efficiently incorporated into VLPs with a mean relative trNGFR amount of 99.6 ± 8.5 ng per 1 µg p24-Gag revealing a Gag to trNGFR mass ratio of approximately 10:1 (Table 1). This equals a calculated average number of 284 ± 24 trNGFR molecules per particle. Thus, VLPs were very efficiently incorporating trNGFR and decorated at similar high densities as reported for HIV-derived viral vector particles [29].

**Figure 2:**
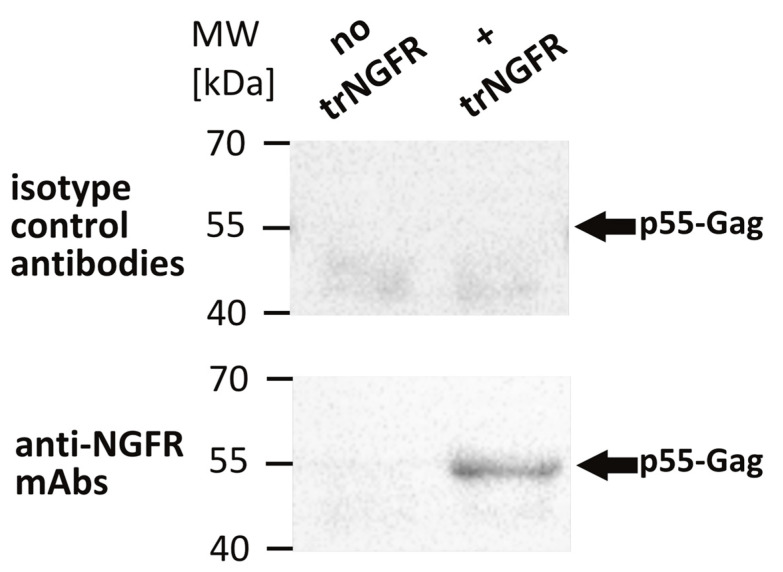
Detection of truncated human nerve growth factor receptor (trNGFR) incorporation in VLPs employing a VLP capture assay followed by an anti-Gag Western blot analysis. VLPs were immunoprecipitated using anti-NGFR mAbs-coated magnetic beads. Beads coated with isotype control antibodies served as negative controls. Samples were subsequently subjected to Western blot analysis using antibodies directed against the p55-Gag precursor protein. Bald VLPs (no trNGFR) were produced by 293-F/Mos1.Gag cells. The trNGFR-VLPs (+ trNGFR) were produced by 293-F/Mos1.Gag/trNGFR cells. The positions of the molecular weight marker (MW) in kilodaltons (kDa) are depicted on the left. Arrows indicate the expected position of p55-Gag proteins.

To confirm the incorporation of trNGFR in VLPs, an NGFR-specific VLP capture assay was performed [26]. Magnetic beads were coated with the anti-NGFR mAb REA844 and isotype control antibodies serving as negative controls, respectively. Bald VLPs produced by 293-F/Mos1.Gag cells as well as presumed trNGFR-displaying VLPs produced by 293-F/Mos1.Gag/trNGFR cells were incubated with the antibody-coated beads. Supernatants and beads were separated in a magnetic field. Beads were thoroughly washed and subjected to Western blot analysis using Gag-specific antibodies to detect trNGFR-positive VLPs. Bald VLPs (no trNGFR) and VLPs precipitated using isotype control antibodies were not observed (Figure 2). In contrast, trNGFR-VLPs were efficiently precipitated using anti-NGFR-coated beads and readily detected in the subsequent Western blot analysis. This proved the incorporation and surface presentation of trNGFR in HIV-derived VLPs. 

### 3.2. Development of a Fixation Protocol for Antigen Preparation

#### 3.2.1. Formaldehyde and Heat Fixation induce a Loss of Epitope Recognition of an Antibody Directed against Native NGFR but Maintain Binding of an FFPE-Compatible Antibody

A fixation protocol applicable to VLPs was developed with the aim of eliciting antibodies directed against FFPE-antigens upon immunization of mice. A common FFPE procedure includes the fixation of cell samples using formaldehyde (typically in a concentration of 4%), subsequent dehydration employing alcohol solutions, clearing in xylene and the final embedding in liquid paraffin at 50 °C to 70 °C. This treatment introduces conformational changes, chemical modification of amino acids, cross-linking of proteins and formation of protein aggregates [6,7]. 

To monitor changes in fixed NGFR, two antibodies with different NGFR recognition profiles were employed. The anti-NGFR mAb C40-1457 is routinely used in flow cytometric analysis and immunohistochemistry (IHC) staining of FFPE tissue sections [30,31]. The epitope recognized by anti-NGFR mAb REA844 in IHC stainings is present in native cells, acetone- and formaldehyde-fixed cells but not in FFPE samples (Miltenyi Biotec, personal communication [32]), and thus not recognizing FFPE-NGFR. Consequently, a fixation procedure needed to be developed resulting in the loss of epitope recognition by mAb REA844 while preserving the reactivity of mAb C40-1457. 

**Figure 3:**
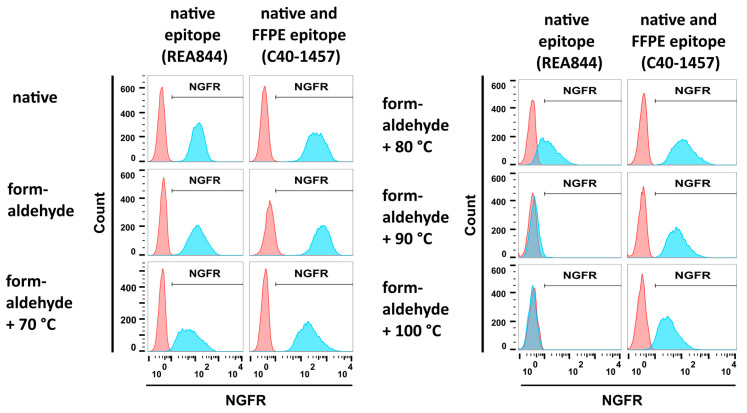
Flow cytometric analysis of mAb reactivities to trNGFR-positive cells upon different fixation procedures. PE-conjugated anti-NGFR mAbs REA844 and C40-1457 (blue) were used to stain 293-F/Mos1.Gag/trNGFR cells. Isotype antibodies served as negative controls (red). REA844 recognizes a native epitope. C40-1457 also recognizes its target epitope upon formaldehyde-fixed paraffin-embedded (FFPE) treatment. Native cells remained untreated. Formaldehyde fixation was performed by incubation in 4% formaldehyde for 1 h. In addition, cells were first fixed in formaldehyde and subsequently incubated for 1 h in PBS at different temperatures as indicated.

293-F/Mos1.Gag/trNGFR cell suspensions were subjected to different fixation steps, incubated with the two anti-NGFR mAbs REA844 and C40-1457 and analyzed employing flow cytometry. Isotype antibodies served as negative controls. Both antibodies bound to native cells and cells fixed using a 4% formaldehyde solution (Figure 3, upper and middle left panels). In the next step and following fixation, cells were incubated in PBS at temperatures ranging from 70 °C to 100 °C. Incubation at 70 °C revealed only a minor effect on NGFR recognition (Figure 3, lower left panel). In contrast, incubation at 80 °C caused a remarkable decrease in native epitope recognition of the mAb REA844 while not altering mAb C40-1457 reactivity (Figure 3, upper right panel). At 90 °C and 100 °C the fluorescent signal intensity of mAb REA844-mediated binding was similar to negative control levels recognition (Figure 3, upper and middle right panels). In general, fluorescence intensities decreased with rising temperatures. However, the FFPE-NGFR-binding mAb C40-1457 still recognized its target on fixed and 90 °C or 100 °C treated cells. Consequently, the combination of formaldehyde fixation and heat treatment at 90 °C (FF90) was chosen for the preparation of VLPs.

#### 3.2.2. VLPs Maintain Their Integrity and Morphology after FF90-Treatment

**Figure 4:**
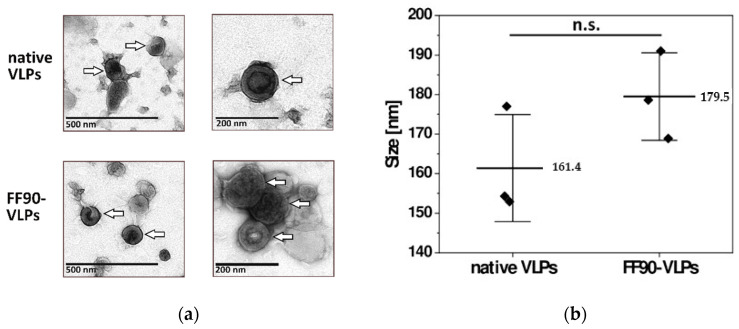
Morphology of VLPs before and after FF90 treatment. (**a**) Negative stain transmission electron microscopic images of native and FF90-VLPs. The arrows indicate VLPs. Scale bars represent 200 nm and 500 nm, respectively. (**b**) Average VLP sizes of native and FF90-trNGFR- VLPs in three independent sample preparations using dynamic light scattering (DLS). Data are shown as mean ± standard deviation. Statistical significance was calculated using an independent two-sample *t*-test (not significant (n.s.): *p* > 0.05).

VLPs for later immunization of mice were subjected to the FF90 fixation procedure and ultracentrifuged to pellet FF90-VLPs. To examine whether FF90-VLPs revealed any morphological changes or signs of disassembly or aggregation after the FF90 treatment, transmission electron microscopic (TEM) imaging and dynamic light scattering (DLS) analysis was performed. TEM imaging of native and FF90-VLPs revealed intact VLPs. No changes in the morphology of either, native or FF90-VLPs, were observed (Figure 4a). VLPs were spherical and displayed the concentric ring of visibly ordered Gag molecules below the VLP membrane, typical for immature particles [33,34]. DLS analysis of three individual VLP preparations demonstrated an average size of native VLPs about 161.4 ± 13.5 nm in diameter. FF90-VLPs were slightly bigger reaching 179.5 ± 11.1 nm (Figure 4b). No significant difference between either values was observed using an independent two-sample t-test (t = −1.793, df = 4, *p* = 0.147). In addition, the samples were monodisperse as indicated by the low polydispersity indices (PdI) of native VLPs (0.110 ± 0.027) and FF90-VLPs (0.163 ± 0.021), i.e., VLPs did not suffer from aggregation upon FF90-treatment. In conclusion, these data demonstrated the very stable and rigid structure of immature HIV-derived VLPs even withstanding exposure to extreme heat.

### 3.3. Immunization of Mice, Generation of Hybridomas and Screening of Monoclonal Antibodies

Hybridoma technology was utilized to generate monoclonal antibodies directed against fixed NGFR. Two mice were immunized intravenously with FF90-VLPs displaying human trNGFR. Three doses of FF90-trNGFR-VLPs (each 9.5 µg total protein) were administered at intervals of 14 days. Two days after the third injection, blood samples were examined for trNGFR-specific antibodies. The first mouse (MZ34) was sacrificed on day 31 and hybridoma cell fusion was conducted. The second mouse (MZ35) received a fourth immunization on day 56 with 3.2 µg total protein of FF90-trNGFR-VLPs. Blood was again analyzed for target-reactive antibodies and splenocytes were isolated and fused with Sp2/0-Ag14 myeloma cells to generate hybridoma cells. 

#### 3.3.1. FF90-trNGFR-VLP Immunization Elicits IgG Antibodies Recognizing FF90-3T3/Gag/trNGFR Cells

**Figure 5:**
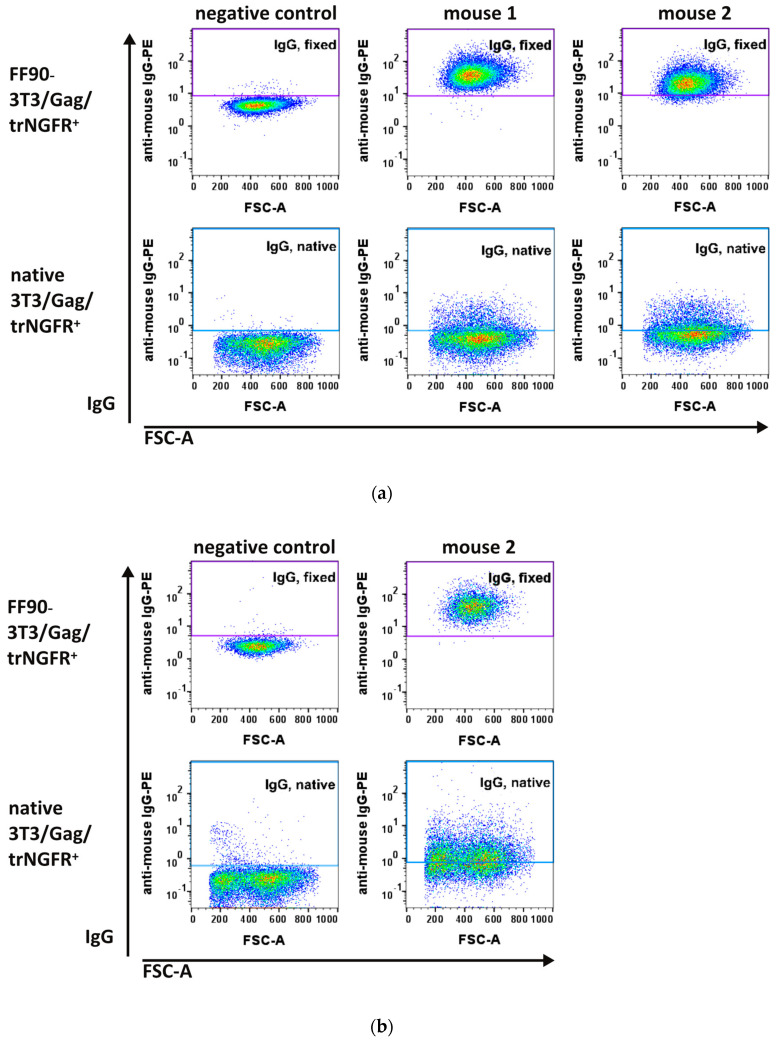
Flow cytometric analysis of IgG antibodies from plasma samples of immunized mice. The 3T3/Gag/trNGFR screening cell line was incubated with plasma samples of bleedings. Bound murine IgG antibodies were subsequently labeled using PE-conjugated anti-mouse IgG1 and IgG2ab secondary antibodies. (**a**) Blood plasma samples taken from mouse 1 and 2 after three immunizations and from a non-immunized mouse serving as negative control. (**b**) Plasma sample from mouse 2 after the fourth immunization with FF90-trNGFR-VLPs.

Two days after the third (MZ34 and MZ35) and fourth immunizations (MZ35), blood samples were taken and examined for antibodies recognizing the human trNGFR-positive murine 3T3/Gag/trNGFR screening cell line. Plasma samples from non-immunized mice served as negative controls. As expected, negative control IgG antibodies did not show specific binding to native and FF90-3T3/Gag/trNGFR cells (Figure 5a, left panels). In contrast, FF90-3T3/Gag/trNGFR cell-reactive IgGs were readily detected in the blood of both mice immunized three times with FF90-trNGFR-VLPs (Figure 5a, upper panels: middle and right) while binding to native target cells was only moderately exceeding the level of the negative control (Figure 5a, lower panels). 

These observations already demonstrated that the immunizations elicited FF90-NGFR-specific antibodies. As the fluorescent intensity of IgG binding in the blood of mouse 1 (MZ34) was slightly more intense as compared to samples of mouse 2 (MZ35), mouse 1 was chosen for the generation of hybridoma cells (Figure 5a, upper panels: middle and right). Mouse 2 received a fourth boosting dose prior to a second bleeding. The additional VLP boost mediated slightly increasing IgG-reactivities to FF90- and native 3T3/Gag/trNGFR cells (Figure 5b). Subsequently, hybridoma cell fusion was performed on day 59.

#### 3.3.2. Hybridoma Cells Produce Antibodies Recognizing FF90-3T3/Gag/trNGFR Cells

Splenocytes fused with Sp2/0-Ag14 myeloma cells generated hybridoma cell cultures showing one or more clusters of cells. Supernatants were screened for IgGs reactive with native and FF90-3T3/Gag/trNGFR cells employing flow cytometry. IgG-producing hybridoma cultures were rated as positive when at least 90% of the native or FF90-treated screening cells were labeled with fluorophore-conjugated anti-mouse IgG1 or IgG2ab secondary antibodies, respectively. Table 2 provides an overview of the screening results. The first hybridoma cell fusion of splenocytes from mouse 1 (MZ34) having received three immunizations yielded a total of 698 hybridoma cell clones. Only three of 698 hybridomas screened produced FF90-3T3/Gag/trNGFR-reactive antibodies (0.4% of tested supernatants). Two mAbs were class IgG1 and one IgG2ab (Table 2). In contrast, hybridomas generated from mouse 2 (MZ35) after four immunizations resulted in 975 clones of which 35 produced FF90-3T3/Gag/trNGFR-reactive mAbs (3.6% of tested supernatants). The majority of mAbs (26) were Ig class IgG2ab and 9 were IgG1 (Table 2). This observation is in accordance with the reported property of VLPs to preferentially elicit an IgG2a antibody response [35,36].

Positive cultures were expanded. Cultures consisting of more than one cell cluster were subcloned using limiting dilution. Hybridoma supernatants were frequently retested during prolonged cultivation. About half of the initial positive clones lost their productivity or stopped proliferating. 15 hybridoma candidates were successfully expanded. They were tested for FF90-NGFR-specificity using NGFR-negative (NGFR⁻) FF90-human peripheral blood mononuclear cells (FF90-PBMCs) and trNGFR-positive FF90-3T3/Gag/trNGFR cells serving as controls.

The trNGFR-VLPs used for immunization were produced in human 293-F/Mos1.Gag/trNGFR cells. Thus, some human host cell proteins were co-incorporated with trNGFR into the VLPs. Human PBMCs were chosen for a 2nd specificity screening round to identify and exclude hybridoma antibodies recognizing human host cell proteins and possibly cross-reacting with homologous antigens present on murine fibroblasts. Stain indices (SIs) were calculated as the difference between the mean fluorescent intensity (MFI) of the tested antibody binding to cognate cells and the MFI resulting from isotype control antibodies divided by 2-fold the standard deviation of the isotype control’s MFI. Antibodies showing a SI > 1 were considered reactive.

**Figure 6:**
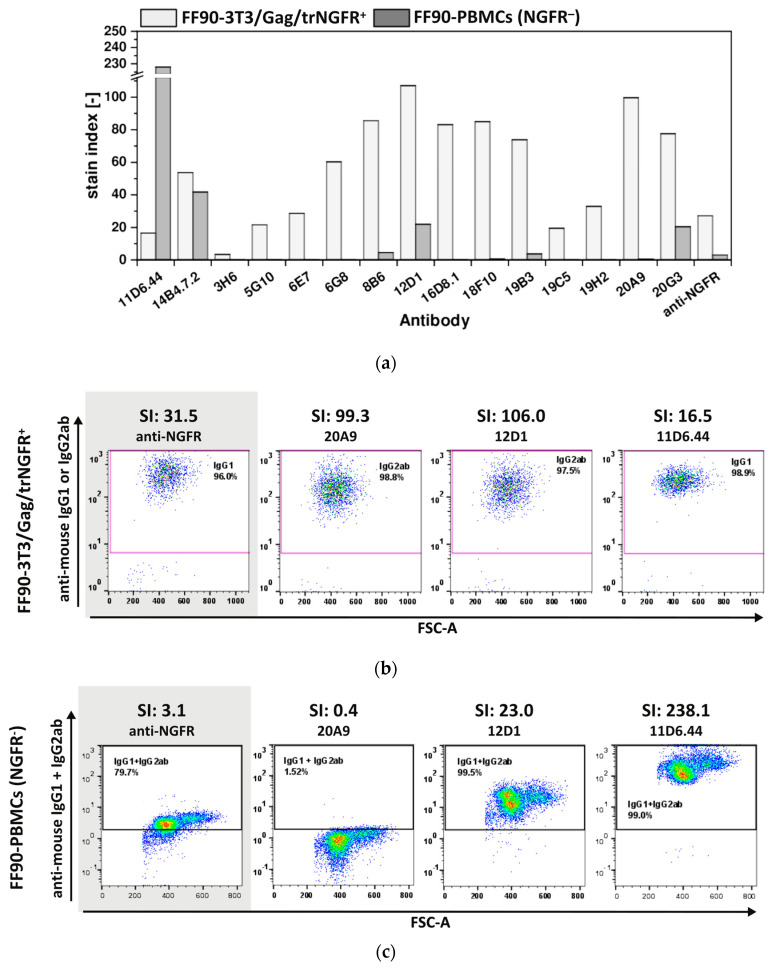
Specificity of mAbs for FF90-NGFR. Human FF90-PBMCs were incubated with either hybridoma supernatants or the control anti-NGFR mAb C40-1457 (anti-NGFR). FF90-3T3/Gag/trNGFR cells stained in parallel with hybridoma-produced mAbs or the anti-NGFR mAb C40-1457 served as positive controls. PE-conjugated anti-mouse IgG1 and IgG2ab served as secondary antibodies. (**a**) Stain indices (SI) of murine antibodies binding FF90-3T3/Gag/trNGFR cells (light gray) and FF90-PBMCs (dark gray). SIs were calculated as the difference between the mean fluorescent intensity (MFI) of the generated mAb and the isotype control MFI divided by 2-fold the standard deviation of the isotype control’s MFI. (**b**) Exemplary FF90-3T3/Gag/trNGFR control cells (trNGFR-positive). The control antibody anti-NGFR mAb C40-1457 is highlighted in gray. (**c**) Exemplary plots of NGFR-negative FF90-PBMCs (NGFR⁻) for four different mAbs and their SI values. The control antibody anti-NGFR mAb C40-1457 is highlighted in gray.

Control staining of FF90-3T3/Gag/trNGFR cells confirmed that all fifteen tested hybridoma antibodies were reactive. Only mAb 3H6 revealed a low reactivity with an SI of 3.5. The SIs of the other 14 mAbs were above 15 (Figure 6a,b). Thus, all fifteen antibodies were demonstrated to be FF90-3T3/Gag/trNGFR binders. In contrast, the SIs of the FF90-PBMCs staining covered a wide range from 0 to 228. The two hybridoma mAbs 11D6.44 and 14B4.7.2 from mouse 1 (MZ34) and four of the thirteen tested mAbs from mouse 2 (MZ35), namely, 8B6, 12D1, 19B3 and 20G3, bound to NGFR-negative FF90-PBMCs (all revealing SIs > 1.0) and therefore were considered non-specific to FF90-NGFR (Table 2, Figure 6a). Noteworthy, the anti-NGFR mAb C40-1457 serving as a positive control exhibited an unspecific binding of NGFR-negative FF90-PBMCs, too, while reaching a much lower SI on FF90-3T3/Gag/trNGFR cells as compared to 8B6 and 19B3. Based on the strict screen criteria applied in this study, mAb C40-1457 would have been considered unspecific for FF90-NGFR. Figure 6c shows representative examples of anti-mouse IgG1 + IgG2 versus forward scatter area (FSC-A) dot plots of FF90-PBMCs at four different SIs.

In summary, no hybridomas derived from mouse 1 (MZ34) were NGFR-specific and nine antibodies, namely 3H6, 5G10, 6E7, 6G8, 16D8.1, 18F10, 19C5, 19H2 and 20A9, stemming from mouse MZ35 showed specificity for FFPE-NGFR as these mAbs did not bind to NGFR-negative FF90-PBMCs. Noteworthy, four of the generated mAbs (6G8, 16D8.1, 18F10, and 20A9) showed not only a much lower background in unspecific binding to FF90-PBMCs but also revealed at least two-fold higher SI values in reactivity to FF90-3T3/Gag/NGFR cells as compared to mAb C40-1457. In conclusion, this demonstrated the high efficiency of FF90-VLP immunization to generate new FF90-antigen-specific mAbs.

#### 3.3.3. The Majority of FF90-NGFR Generated mAbs Enables Also the Detection of FFPE-NGFR

**Figure 7:**
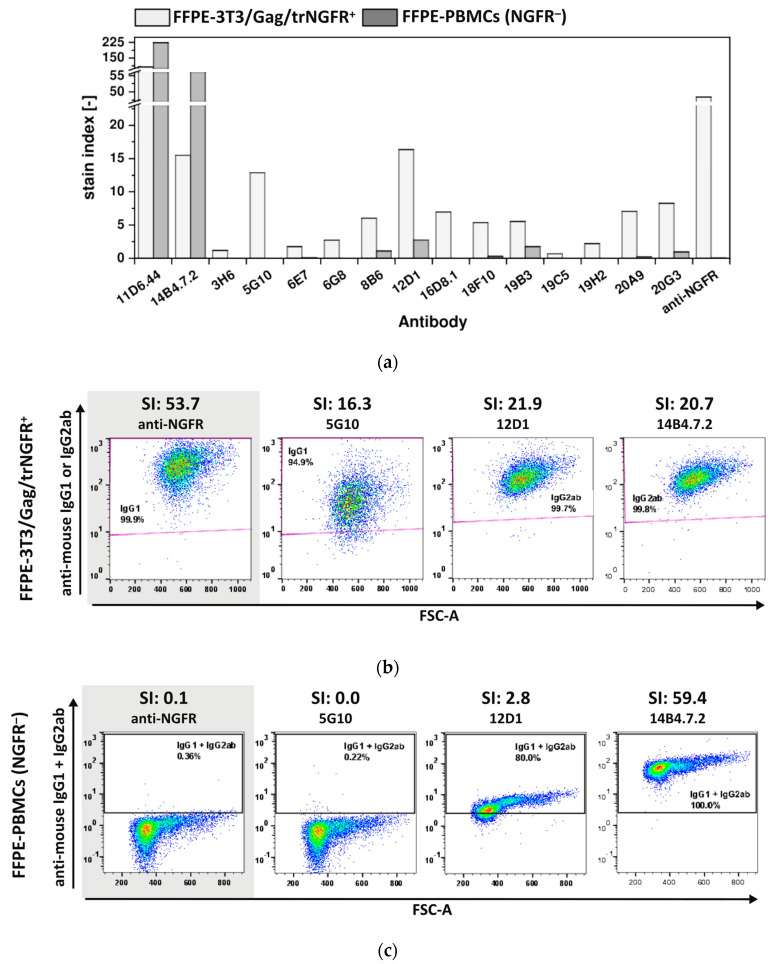
Reactivity and specificity of mAbs for FFPE-NGFR prepared with an alternative fixation protocol. FFPE-3T3/Gag/trNGFR cells were stained with generated antibodies or the anti-NGFR mAb C40-1457 to identify mAbs recognizing FFPE-NGFR. In parallel, human NGFR-negative FFPE-PBMCs were stained with novel and control mAbs. PE-conjugated anti-mouse IgG1 and IgG2ab served as secondary antibodies. (**a**) Stain indices (SIs) of murine mAbs upon staining of FFPE-3T3/Gag/trNGFR cells (light gray) and FFPE-PBMCs (dark gray). SIs were calculated as the difference between the mean fluorescent intensity (MFI) of the generated mAb and the isotype control MFI divided by 2-fold the standard deviation of the isotype control’s MFI. (**b**) Exemplary FFPE-3T3/Gag/trNGFR control cells. The control antibody anti-NGFR mAb C40-1457 is highlighted in gray. (**c**) Exemplary plots of NGFR-negative FFPE-PBMCs for four different mAbs and their SI values. The control antibody anti-NGFR mAb C40-1457 is highlighted in gray.

The ultimate aim of this POC study was to generate mAbs recognizing FFPE-NGFR, as samples and tissues preserved by FFPE treatment represent a huge source of material for research studies provided that FFPE-antigen-specific mAbs are available [3]. To investigate whether the generated novel mAbs also enabled detection of FFPE-NGFR, 3T3/Gag/trNGFR cells and PBMCs were fixed following the protocol for FFPE single cell preparation as described in the material and method section. FFPE-cells were employed to examine the 15 FF90-3T3/Gag/trNGFR binders for their reactivity and specificity to FFPE-NGFR (Figure 7).

Both hybridoma antibodies, 11D6.44 and 14B4.7.2, generated in mouse 1 (MZ34) and initially identified as FF90-3T3/Gag/trNGFR binders recognized FFPE-3T3/Gag/trNGFR cells (Figure 7a, Table 3). Furthermore, 12 of 13 mAbs from mouse 2 (MZ35) were identified as FFPE-3T3/Gag/trNGFR binders (92.3% of FF90-3T3/Gag/trNGFR-reactive mAbs; Figure 7a, Table 3). The SIs of the generated mAbs using FFPE-3T3/Gag/trNGFR target cells were generally lower than the cognate SIs of FF90-3T3/Gag/trNGFR stainings and ranged from 0.7 to 107.4 (Figure 6a and Figure 7a). The mAb 19C5 exhibited an SI of 0.7 and was therefore classified as an FFPE non-binder. The other mAbs had SIs > 1.0 and were consequently rated reactive to FFPE-3T3/Gag/trNGFR cells. The anti-NGFR positive control mAb C40-1457 reached an SI of 53.7 using FFPE-3T3/Gag/trNGFR cells. Figure 7b shows exemplary dot plots of mAbs binding to FFPE-3T3/Gag/trNGFR cells. 

SIs of FFPE-PBMC staining ranged from 0.0 to 223.6 with the majority of SIs < 1.0. The mAbs 11D6.44 and 14B4.7.2 generated in mouse 1 (MZ34, only three immunizations) as well as 8B6, 12D1 and 19B3 generated in mouse 2 (MZ35) upon four immunizations revealed SIs > 1.0 indicating non-specificity for FFPE-NGFR (Figure 7a, Table 3). In contrast to the previous observation of FF90-PBMCs labeling, the anti-NGFR control mAb C40-1457 did not bind to FFPE-PBMCs. Figure 7c shows representative examples of anti-mouse IgG1 + IgG2 versus FSC-A dot plots of FFPE-PBMCs at different SIs.

To characterize the described hybridoma cells in more detail, mRNA will be isolated and full-length heavy and light chain encoding sequences will be amplified using RT-PCR. DNA sequencing will reveal how many different mAbs were generated and isolated. However, considering the very distinguished binding profiles, SIs and that antibodies of classes IgG1 and IgG2 were generated, it appears feasible to assume that most if not all hybridoma clones were producing individual mAbs. 

In summary, mAbs derived from mouse 1 (MZ34) were not FFPE-NGFR-specific, but nine mAbs derived from mouse 2 (MZ35) after a fourth additional immunization revealed specificity for FFPE-NGFR, namely 3H6, 5G10, 6E7, 6G8, 16D8.1, 18F10, 19H2, 20A9 and 20G3. This demonstrated that the FF90 fixation facilitates the elicitation of mAbs also reactive to FFPE-target antigens. Considering the dense decoration of the VLPs with the target antigen trNGFR and the insufficiency of three immunizations to generate FFPE-target-specific mAbs, this could suggest that the immunogenicity of antigen-decorated VLPs was strongly impacted by fixation. This warrants further comparative studies using FF90- and native VLPs displaying a variety of surface antigens, employing different immunization schemes and routes, possibly higher doses and also the use of adjuvants. However, we anticipate that this POC and the simplified fixation protocol FF90 will be of utility in future antibody discovery studies to identify FFPE-antigen-reactive and -specific mAbs.

## Figures and Tables

**Table 1:** NGFR quantitation in CFSNs harvested from 293-F, 293-F/Mos1.Gag and 293-F/Mos1.Gag/trNGFR cells. NGFR concentrations were detected using an NGFR-specific ELISA and samples normalized for p24-Gag concentrations. Data shown represent mean ± standard deviation of biological triplicates.

Cells	NGFR per 1 µg Gag	Number of Replicates
293-F	<0.08 ng	n = 1
293-F/Mos1.Gag	<0.08 ng	n = 3
293-F/Mos1.Gag/trNGFR	99.6 ± 8.5 ng	n = 3

**Table 2:** Summary of the flow cytometric analysis of hybridoma-produced antibodies for FF90-NGFR-reactivity and -specificity. In the first screen, antibodies were tested for reactivity with native and FF90-3T3/Gag/trNGFR cells. In the second screening, hybridoma supernatants were tested for specificity to the target antigen employing NGFR-negative FF90-treated human peripheral blood mononuclear cells (FF90-PBMCs).

Immunization (ID)	Screen	Screening Cells	Binding mAbs/ Tested mAbs	IgG subclass
3 x FF90-trNGFR-VLPs (MZ34)	1st	native and FF90-3T3/Gag/trNGFR^+^	3/698 (0.4%) ^1^	1 × IgG1; 2 × IgG2ab
	2nd	FF90-PBMCs (NGFR⁻)	2/2 (100%) ^2^	
4 x FF90-trNGFR-VLPs (MZ35)	1st	native and FF90-3T3/Gag/trNGFR^+^	35/975 (3.6%) ^1^	9 × IgG1; 26 × IgG2ab
	2nd	FF90-PBMCs (NGFR⁻)	4/13 (30.8%) ^2^	

^1^ Ratio of number of positive hybridoma supernatants to total number of screened supernatants. ^2^ Ratio of number of NGFR-unspecific hybridoma supernatants to 1st screening positive ones.

**Table 3:** Summary of the flow cytometric analysis of mAbs for FFPE-NGFR reactivity and specificity. Fifteen mAbs binding FF90-3T3/Gag/trNGFR cells were tested for reactivity with FFPE-3T3/Gag/trNGFR cells and FFPE-PBMCs (NGFR^−^) fixed using an alternative fixation protocol.

Immunization (ID)	Screening Cells	Binding mAbs/ Tested mAbs
3 x FF90-trNGFR-VLPs (MZ34)	FFPE-3T3/Gag/trNGFR+	2/2 (100%) ^1^
	FFPE-PBMCs (NGFR^−^)	2/2 (100%) ^2^
4 x FF90-trNGFR-VLPs (MZ35)	FFPE-3T3/Gag/trNGFR+	12/13 (92.3%) ^1^
	FFPE-PBMCs (NGFR^−^)	3/12 (25.0%) ^2^

^1^ Ratio of number of reactive mAbs and total number of mAbs identified as FF90-3T3/Gag/trNGFR binders. ^2^ Ratio of number of NGFR-unspecific mAbs and total number of mAbs identified as FFPE-3T3/Gag/trNGFR binders.

## Data Availability

Data sharing not applicable. No new data were created or analyzed in this study. Data sharing is not applicable to this article.

## Supplementary Materials

The following supporting information can be downloaded at: https://www.mdpi.com/article/10.3390/antib12030057/s1, Figure S1: Schematic illustration of the transposase expression constructs and the transposon donor vectors.

## References

[1] Köhler G., Milstein C. (1975). Continuous cultures of fused cells secreting antibody of predefined specificity. Nature.

[2] Meneses-Acosta A., Gómez A., Ramírez O.T. (2012). Control of redox potential in hybridoma cultures: Effects on MAb production, metabolism, and apoptosis. J. Ind. Microbiol. Biotechnol..

[3] Gaffney E.F., Riegman P.H., Grizzle W.E., Watson P.H. (2018). Factors that drive the increasing use of FFPE tissue in basic and translational cancer research. Biotech. Histochem. Off. Public Biol. Stain. Comm..

[4] Ramos-Vara J.A., Miller M.A. (2014). When tissue antigens and antibodies get along: Revisiting the technical aspects of immunohistochemistry-the red, brown, and blue technique. Vet. Pathol..

[5] Haverkamp A.-K., Bosch B.J., Spitzbarth I., Lehmbecker A., Te N., Bensaid A., Segalés J., Baumgärtner W. (2019). Detection of MERS-CoV antigen on formalin-fixed paraffin-embedded nasal tissue of alpacas by immunohistochemistry using human monoclonal antibodies directed against different epitopes of the spike protein. Vet. Immunol. Immunopathol..

[6] Werner M., Chott A., Fabiano A., Battifora H. (2000). Effect of formalin tissue fixation and processing on immunohistochemistry. Am. J. Surg. Pathol..

[7] Thavarajah R., Mudimbaimannar V.K., Elizabeth J., Rao U.K., Ranganathan K. (2012). Chemical and physical basics of routine formaldehyde fixation. J. Oral Maxillofac. Pathol. JOMFP.

[8] Soares H.R., Castro R., Tomás H.A., Rodrigues A.F., Gomes-Alves P., Bellier B., Klatzmann D., Carrondo M.J.T., Alves P.M., Coroadinha A.S. (2016). Tetraspanins displayed in retrovirus-derived virus-like particles and their immunogenicity. Vaccine.

[9] Schneider I.C., Hartmann J., Braun G., Stitz J., Klamp T., Bihi M., Sahin U., Buchholz C.J. (2018). Displaying Tetra-Membrane Spanning Claudins on Enveloped Virus-Like Particles for Cancer Immunotherapy. Biotechnol. J..

[10] Sailaja G., Skountzou I., Quan F.-S., Compans R.W., Kang S.-M. (2007). Human immunodeficiency virus-like particles activate multiple types of immune cells. Virology.

[11] McFall-Boegeman H., Huang X. (2022). Mechanisms of cellular and humoral immunity through the lens of VLP-based vaccines. Expert Rev. Vaccines.

[12] Mátés L., Chuah M.K.L., Belay E., Jerchow B., Manoj N., Acosta-Sanchez A., Grzela D.P., Schmitt A., Becker K., Matrai J. (2009). Molecular evolution of a novel hyperactive Sleeping Beauty transposase enables robust stable gene transfer in vertebrates. Nat. Genet..

[13] Yusa K., Zhou L., Li M.A., Bradley A., Craig N.L. (2011). A hyperactive piggyBac transposase for mammalian applications. Proc. Natl. Acad. Sci. USA.

[14] Eggenschwiler R., Gschwendtberger T., Felski C., Jahn C., Langer F., Sterneckert J., Hermann A., Lühmann J., Steinemann D., Haase A. (2021). A selectable all-in-one CRISPR prime editing piggyBac transposon allows for highly efficient gene editing in human cell lines. Sci. Rep..

[15] Yang A.G., Zhang X., Torti F., Chen S.Y. (1998). Anti-HIV type 1 activity of wild-type and functional defective RANTES intrakine in primary human lymphocytes. Hum. Gene Ther..

[16] Castellino S.M., Kurtzberg J., Smith C. (1999). Retroviral vector-mediated gene transfer into umbilical cord blood-derived megakaryocyte and platelet progenitors. Biol. Blood Marrow Transplant..

[17] Langedijk J.P.M., van Manen D., Vellinga J., Wegmann F., Callendret B.C.S., Krarup A., Stitz J. (2019). Synthetic Human Immunodeficiency Virus (HIV) Envelope Antigen, Vectors, and Compositions Thereof. U.S. Patent.

[18] Rosengarten J.F., Schatz S., Wolf T., Barbe S., Stitz J. (2022). Components of a HIV-1 vaccine mediate virus-like particle (VLP)-formation and display of envelope proteins exposing broadly neutralizing epitopes. Virology.

[19] Berg K., Schäfer V.N., Tschorn N., Stitz J. (2020). Advanced Establishment of Stable Recombinant Human Suspension Cell Lines Using Genotype-Phenotype Coupling Transposon Vectors. Methods Mol. Biol..

[20] Berg K., Schäfer V.N., Bartnicki N., Eggenschwiler R., Cantz T., Stitz J. (2019). Rapid establishment of stable retroviral packaging cells and recombinant susceptible target cell lines employing novel transposon vectors derived from Sleeping Beauty. Virology.

[21] Sharma N., Hollensen A.K., Bak R.O., Staunstrup N.H., Schrøder L.D., Mikkelsen J.G. (2012). The impact of cHS4 insulators on DNA transposon vector mobilization and silencing in retinal pigment epithelium cells. PLoS ONE.

[22] Ivics Z., Hackett P.B., Plasterk R.H., Izsvák Z. (1997). Molecular Reconstruction of Sleeping Beauty, a Tc1-like Transposon from Fish, and Its Transposition in Human Cells. Cell.

[23] Lynch A., Meyers A.E., Williamson A.-L., Rybicki E.P. (2012). Stability studies of HIV-1 Pr55gag virus-like particles made in insect cells after storage in various formulation media. Virol. J..

[24] Maecker H.T., Frey T., Nomura L.E., Trotter J. (2004). Selecting fluorochrome conjugates for maximum sensitivity. Cytom. Part A.

[25] Lavado-García J., Jorge I., Boix-Besora A., Vázquez J., Gòdia F., Cervera L. (2021). Characterization of HIV-1 virus-like particles and determination of Gag stoichiometry for different production platforms. Biotechnol. Bioeng..

[26] Rosengarten J.F., Schatz S., Stitz J. (2022). Detection of Neutralization-sensitive Epitopes in Antigens Displayed on Virus-Like Particle (VLP)-Based Vaccines Using a Capture Assay. J. Vis. Exp..

[27] Schatz S., van Dijk F.H., Dubiel A.E., Cantz T., Eggenschwiler R., Stitz J., Zielonka S., Krah S. (2023). Generation of Human 293-F Suspension NGFR Knockout Cells Using CRISPR/Cas9 Coupled to Fluorescent Protein Expression. Genotype Phenotype Coupling: Methods and Protocols.

[28] Sharma D., Barhwal K.K., Biswal S.N., Srivastava A.K., Bhardwaj P., Kumar A., Chaurasia O.P., Hota S.K. (2019). Hypoxia-mediated alteration in cholesterol oxidation and raft dynamics regulates BDNF signalling and neurodegeneration in hippocampus. J. Neurochem..

[29] Jamali A., Kapitza L., Schaser T., Johnston I.C.D., Buchholz C.J., Hartmann J. (2019). Highly Efficient and Selective CAR-Gene Transfer Using CD4- and CD8-Targeted Lentiviral Vectors. Mol. Ther.-Methods Clin. Dev..

[30] Fujii K., Morita S., Mochizuki M., Shibuya-Takahashi R., Fujimori H., Yamaguchi K., Abe J., Yamazaki T., Imai T., Sugamura K. (2022). Establishment of a monoclonal antibody against glycosylated CD271 specific for cancer cells in immunohistochemistry. Cancer Sci..

[31] Lin J.-R., Izar B., Wang S., Yapp C., Mei S., Shah P.M., Santagata S., Sorger P.K. (2018). Highly multiplexed immunofluorescence imaging of human tissues and tumors using t-CyCIF and conventional optical microscopes. eLife.

[32] Johnston I.C.D. (2021). List of anti-NGFR mAbs tested for FFPE tissue reactivity: The anti-NGFR mAb REA844 (Miltenyi Biotec, Germany) is not reactive with NGFR in FFPE tissue samples. Personal communication.

[33] Wright E.R., Schooler J.B., Ding H.J., Kieffer C., Fillmore C., Sundquist W.I., Jensen G.J. (2007). Electron cryotomography of immature HIV-1 virions reveals the structure of the CA and SP1 Gag shells. EMBO J..

[34] Martin J.L., Cao S., Maldonado J.O., Zhang W., Mansky L.M. (2016). Distinct Particle Morphologies Revealed through Comparative Parallel Analyses of Retrovirus-Like Particles. J. Virol..

[35] Zhang S., Cubas R., Li M., Chen C., Yao Q. (2009). Virus-like particle vaccine activates conventional B2 cells and promotes B cell differentiation to IgG2a producing plasma cells. Mol. Immunol..

[36] Lee Y., Lee Y.-T., Ko E.-J., Kim K.-H., Hwang H.S., Park S., Kwon Y.-M., Kang S.M. (2017). Soluble F proteins exacerbate pulmonary histopathology after vaccination upon respiratory syncytial virus challenge but not when presented on virus-like particles. Hum. Vaccines Immunother..

