# Supplementary material for: Generation of Antibodies Selectively Recognizing Epitopes in a Formaldehyde-Fixed Cell-Surface Antigen Using Virus-like Particle Display and Hybridoma Technology
Source: Antibodies (Basel). 2023 Sep 5;12(3):57. doi: 10.3390/antib12030057 (PMC10525569; doi:10.3390/antib12030057)
Supplement: Supplementary file 1 [file antibodies-12-00057-s001.zip › antibodies-2575240-SI.pdf]

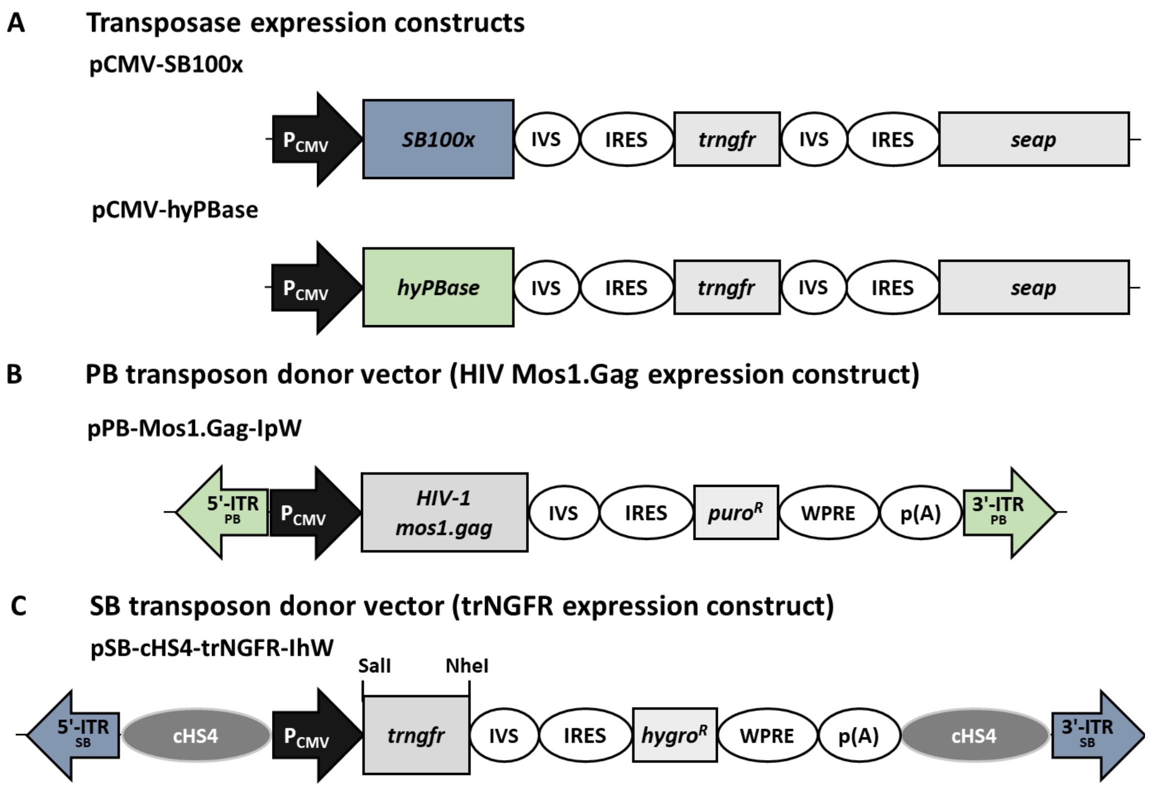


**Figure S1. Schematic illustration of the transposase expression constructs and the transposon donor vectors.** The expression of the genes of interest in all constructs is driven by the CMV promoter/enhancer element ($P_{CMV}$). **A** The transposase expression constructs pCMV-hyPBase and pCMV-SB100x mediate the transient expression of the hyperactive *piggyBac* (PB) transposase (hyPBase) and the hyperactive *Sleeping Beauty* (SB) transposase variant SB100x, respectively. A synthetic intron (IVS) and an internal ribosomal entry site (IRES) couple the expression of the transposases to the expression of the two reporter genes truncated nerve growth factor receptor (*trngfr*) and secreted alkaline phosphatase (*seap*). **B** The PB transposon donor vector pPB-Mos1.Gag-IpW encodes for the HIV-1 mosaic Gag (Mos1.Gag) protein. The expression of Mos1.Gag is coupled to the expression of a puromycin resistance gene (*puro*$^R$). The expression cassette ends with the woodchuck hepatitis posttranscriptional regulatory element (WPRE) and the polyadenylation signal p(A) of the bovine growth hormone. The expression cassette is flanked by PB-derived inverted terminal repeats (ITRs). **C** The SB transposon donor vector pSB-cHS4-trNGFR-IhW encodes for the human truncated nerve growth factor receptor (trNGFR) coupled to the expression of a hygromycin resistance gene (*hygro*$^R$). The expression cassette ends with the woodchuck hepatitis posttranscriptional regulatory element (WPRE) and the polyadenylation signal p(A) of the bovine growth hormone. The expression cassette is flanked by two copies of the core chicken beta-globulin insulator sequences (cHS4) and SB-derived inverted terminal repeats (ITRs).
